# Age-Related Changes in the Cellular Composition and Epithelial Organization of the Mouse Trachea

**DOI:** 10.1371/journal.pone.0093496

**Published:** 2014-03-27

**Authors:** Carolien Wansleeben, Emily Bowie, Danielle F. Hotten, Yen-Rei A. Yu, Brigid L. M. Hogan

**Affiliations:** 1 Department of Cell Biology, Duke University Medical Centre, Durham, North Carolina, United States of America; 2 Department of Medicine, Duke University Medical Centre, Durham, North Carolina, United States of America; University of Pennsylvania School of Medicine, United States of America

## Abstract

We report here senescent changes in the structure and organization of the mucociliary pseudostratified epithelium of the mouse trachea and main stem bronchi. We confirm previous reports of the gradual appearance of age-related, gland-like structures (ARGLS) in the submucosa, especially in the intercartilage regions and carina. Immunohistochemistry shows these structures contain ciliated and secretory cells and Krt5+ basal cells, but not the myoepithelial cells or ciliated ducts typical of normal submucosal glands. Data suggest they arise de novo by budding from the surface epithelium rather than by delayed growth of rudimentary or cryptic submucosal glands. In old mice the surface epithelium contains fewer cells per unit length than in young mice and the proportion of Krt5+, p63+ basal cells is reduced in both males and females. However, there appears to be no significant difference in the ability of basal stem cells isolated from individual young and old mice to form clonal tracheospheres in culture or in the ability of the epithelium to repair after damage by inhaled sulfur dioxide. Gene expression analysis by Affymetrix microarray and quantitative PCR, as well as immunohistochemistry and flow sorting studies, are consistent with low-grade chronic inflammation in the tracheas of old versus young mice and an increase in the number of immune cells. The significance of these changes for ARGL formation are not clear since several treatments that induce acute inflammation in young mice did not result in budding of the surface epithelium.

## Introduction

Recent studies in a variety of epithelial tissues have shown that aging is associated with a loss of homeostasis and alterations in stem cells and their niches. In some cases these modifications correlate with a decline in tissue function, for example reduced wound repair in the epidermis of the mouse skin [1], defective regeneration of exocrine and endocrine pancreas [2], [3] and reduced differentiation of stem cells in the Drosophila midgut [4], [5]. In the case of the lungs, aging in both humans and rodents is associated with a variety of structural and pathologic changes. These changes include airspace enlargement, decreased lung compliance, and increased risk for respiratory disorders such as chronic obstructive pulmonary disease (COPD), emphysema, submucosal gland hypertrophy and idiopathic pulmonary fibrosis (IPF), as well as alterations in the innate immune system and low-grade chronic inflammation [6]–[11]. However, the underlying cellular mechanisms responsible for age-related changes in the phenotype of the respiratory epithelium are poorly understood, hindering novel therapeutic approaches.

The trachea and main stem bronchi of the mouse lung, and most of the intralobar airways of the human lung, are lined by a pseudostratified mucociliary epithelium [12]. This contains mainly ciliated cells and different classes of secretory cells (serous, club/Clara and goblet cells) that change in their proportion along the proximal-distal axis. In addition, the epithelium contains a population of basal cells that express p63 and cytokeratin 5 (Krt5) and function as multipotent stem cells capable of long term self-renewal and differentiation into multiciliated and secretory cells [13], [14]. The airways of the human lung also contain numerous submucosal glands (SMGs). These are composed of acini with serous and mucus secretory cells and myoepithelial basal cells. They are connected to the main airways by ducts lined by multiciliated cells and basal cells [15], [16]. In the young mouse, SMGs are confined to the most proximal part of the trachea and extralobar bronchi. However, in 1970 Nettesheim and Martin reported the presence in old mice of numerous epithelial cysts in the submucosal tissue underlying the lumen of the distal trachea and extralobar bronchi. Small clusters of these age-related gland-like structures (ARGLS) were seen at 7 months and they increased in number up to 2 years [17]. In some of the oldest mice, a nearly continuous layer of ARGLS, typically filled with cell debris, crystals and PAS-positive material, was found in the carina, which in younger mice is completely devoid of glands. We have confirmed these findings and provide evidence that ARGLS likely arise by de novo budding of cells from the surface epithelium rather than from the growth and expansion of cryptic glands present in the submucosa from birth. In addition, we report a decrease in the number and proportion of basal cells in the epithelium lining the airways. Global transcriptome analysis and flow cytometric data provide evidence for changes in gene expression in the aging trachea and an increase in the number of activated B and T cells; these parameters are consistent with the development of low grade chronic inflammation. Taken together, our findings indicate that senescence of the mouse lung is associated with numerous changes in the cellular composition, organization and local microenvironment of the epithelium lining the upper airways.

## Results

### Appearance of age-related gland like structures in mouse proximal airways

**Figure 1:**
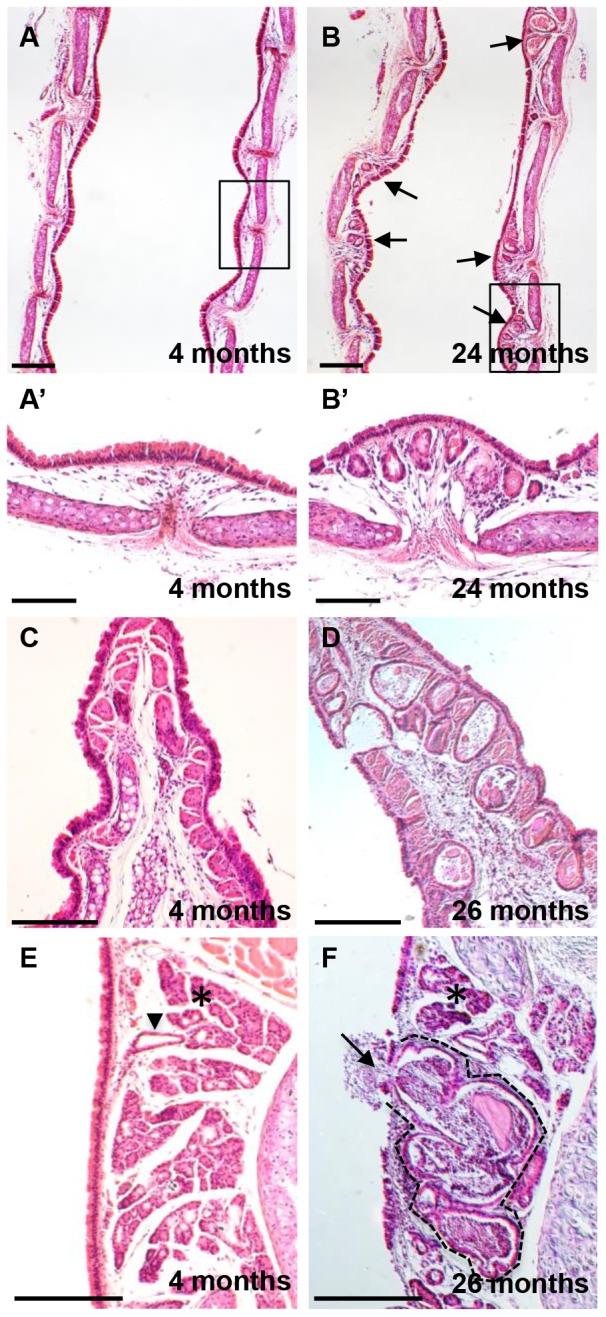
Age-related changes in the organization of tracheal epithelium. Longitudinal midline sections of tracheas from young (4–7month) and old (24–26 month) male C57Bl/6 mice were stained with haematoxylin and eosin. (A, B) Low power view showing ARGLS in the intercartilage regions (arrows). (A′, B′) High power view of boxed regions. (C, D) Note high density of ARGLS in the carina of an old mouse and the direct connection between an ARGL and the airway lumen. (E, F) Section through distal trachea showing an ARGLS (dotted line) within a submucosal gland. The cystic structure is engorged with extracellular material and there is apparent discharge of this material into the airway lumen (arrow). By contrast normal glands (asterix) are not enlarged and are connected to the airway lumen by ducts (arrowhead) Scale bars: A–D  = 500 μm; E–G  = 100 μm.

Histology confirmed the presence of gland-like structures (ARGLS) in the submucosa underlying the entire trachea and main stem bronchi of old mice (Fig. 1 and data not shown for bronchi) [17]. The structures are most frequent in the intercartilage regions and carina, and absent from the intralobar airways. We also found ARGLS intermingled with normal SMGs in the proximal trachea (Fig 1F). Significantly, ARGLS were never seen to connect to the surface epithelium by ducts lined by multiciliated cells, a feature typical of SMGs (Fig. 1E); rather it appears that their contents could be released directly into the tracheal lumen (Fig. 1F). Table 1 summarizes observations on a total of 35 C57Bl/6 mice of different ages and different commercial sources. We conclude that ARGLSs appear around 5–7 months of age, with no significant difference in abundance between males and females or mice purchased from different commercial sources.

**Table 1:** Age dependence of ARGLs.

Age C57BL/6 (♂and ♀)	Proximal trachea	Distal trachea
4 months	−	−
7–10 months	+	+
12–16 months	++	+++
24–28 months	+++	+++++

Presence of ARGLs in longitudinal midline sections of entire trachea and main stem bronchi of male and female C57Bl/6 mice aged 4 (n = 10), 7–10 (n = 10), 12–16 (n = 5) and 24–28 (n = 10) months. - no ARGLs, + 1–2 ARGLs/section, ++ 3–6 ARGLs/section, +++ 6–8 ARGLs/section and ++++ more than 8 ARGLS/section. No difference was noted between males and females or between mice from difference sources.

### Cellular composition of ARGLS

**Figure 2:**
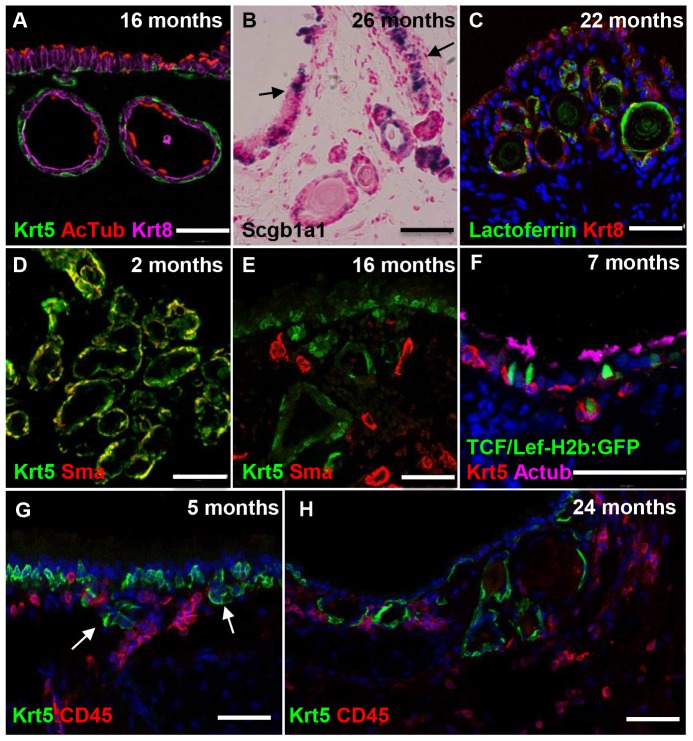
Phenotype and development of Age-Related Gland-Like Structures. Sections of tracheas of mice between 2 and 24 months of age were analyzed by immunohistochemistry (A, C–H) and in situ hybridization (B). (A) Section of 16 month old trachea showing Krt5+ basal cells (green) and AcTub+ multiciliated cells (red) in both surface epithelium and ARGLS. (B) 26 month old trachea showing secretory cells that express *Scgb1a1* RNA in both ARGLS and tracheal epithelium (arrows). (C) Section of 22 month old trachea showing lactoferrin + Krt8+ cells in both ARGLs and surface epithelium. (D) Myoepithelial cells in the acini of a 2 month old submucosal gland are positive for both Krt5 (green) and smooth muscle actin (sma) (red). (E) Basal cells in 16 month old ARGLS express Krt5 but not smooth muscle actin, which is only seen in adjacent blood vessels. (F) Section of 7 month old trachea of a TCF-LEF-H2b:GFP transgenic mouse showing a small bud that likely represents a newly forming ARGLS. A cell in the bud expresses both Krt5 and H2b:GFP. (G) Section of 5 month trachea showing small clusters of Krt5+ cells (green) below the surface epithelium. CD45+ immune cells are present close to the clusters (red) (H) Section of 24 month old trachea showing presence of CD45+ immune cells in the stroma around ARGLS that contain Krt5+ basal cells (green). Scale bars: A–E 50 μm, F–H 100 μm.

As described previously [17] ARGLS are lined by cuboidal epithelium and contain cellular debris and crystalline and proteinaceous material (Fig. 1 and data not shown). Nettesheim and Martin offered no explanation for the origin of these structures. However, based on their higher density in intercartilage regions, where SMGs reside in the proximal trachea, one possibility is that they are derived from preexisting, but very rudimentary or cryptic SMGs that grow late in life. Alternatively, they may develop by de novo budding or delamination of the aging luminal epithelium into the underlying mesenchyme, in which case their cellular composition would be expected to resemble that of the tracheal epithelium rather than SMGs. We therefore used immunohistochemistry to examine ARGLs in more detail. This revealed that the structures contain multiciliated cells as well as secretory cells that are positive for Scgb1a1 transcripts and lactoferrin protein, markers expressed in both SMGs and luminal epithelium (Fig. 2A–C). Both SMG and surface epithelium contain basal cells that express p63 and Krt5. However, the basal cells in the acini of SMGs also co-express high levels of smooth muscle actin, a feature typical of myoepithelial cells (Fig. 2D). Significantly, none of the p63+ Krt5+ basal cells in ARGLs express smooth muscle actin, even though nearby smooth muscle cells in the stroma are positive (Fig. 2E).

In the course of staining sections with antibodies to Krt5 we observed a few small clusters of cells underneath the surface epithelium in intercartilage regions in mice 5 months of age (Fig. 2G). The number and size of these clusters increased with age. Further analysis showed that some of the Krt5+ cells express GFP from the TCF/Lef-H2b:GFP reporter allele [18], suggesting that they are responding to canonical Wnt signaling (Fig. 2F). Taken together, our results support a model in which ARGLSs arise by the budding or delamination of Krt5+ cells from the tracheal epithelium into the underlying mesenchyme rather than from preexisting but rudimentary SMGs initially laid down soon after birth.

### ARGLSs in mutant mice

A previous study had reported the presence of submucosal gland-like structures in the distal trachea of *Myd88* null mutant mice compared with wild type [19] although the age of the mutant mice was not recorded. We therefore examined *Myd88* null mice on the C57Bl/6 genetic background at 2.5, 8.5 and 12.5 months of age (n = 1 at each age). However, we failed to find any histological differences in the tracheas of mutant versus wild type mice of the same age on the same genetic background. We also examined the tracheas of young *Ob/Ob* homozygous mutants because obesity has been reported to exacerbate the response of lungs to environmental agents [20]. However, we again found no evidence for premature ARGL formation.

### Basal cells and reparative ability of tracheal epithelium of young versus old mice

**Figure 3:**
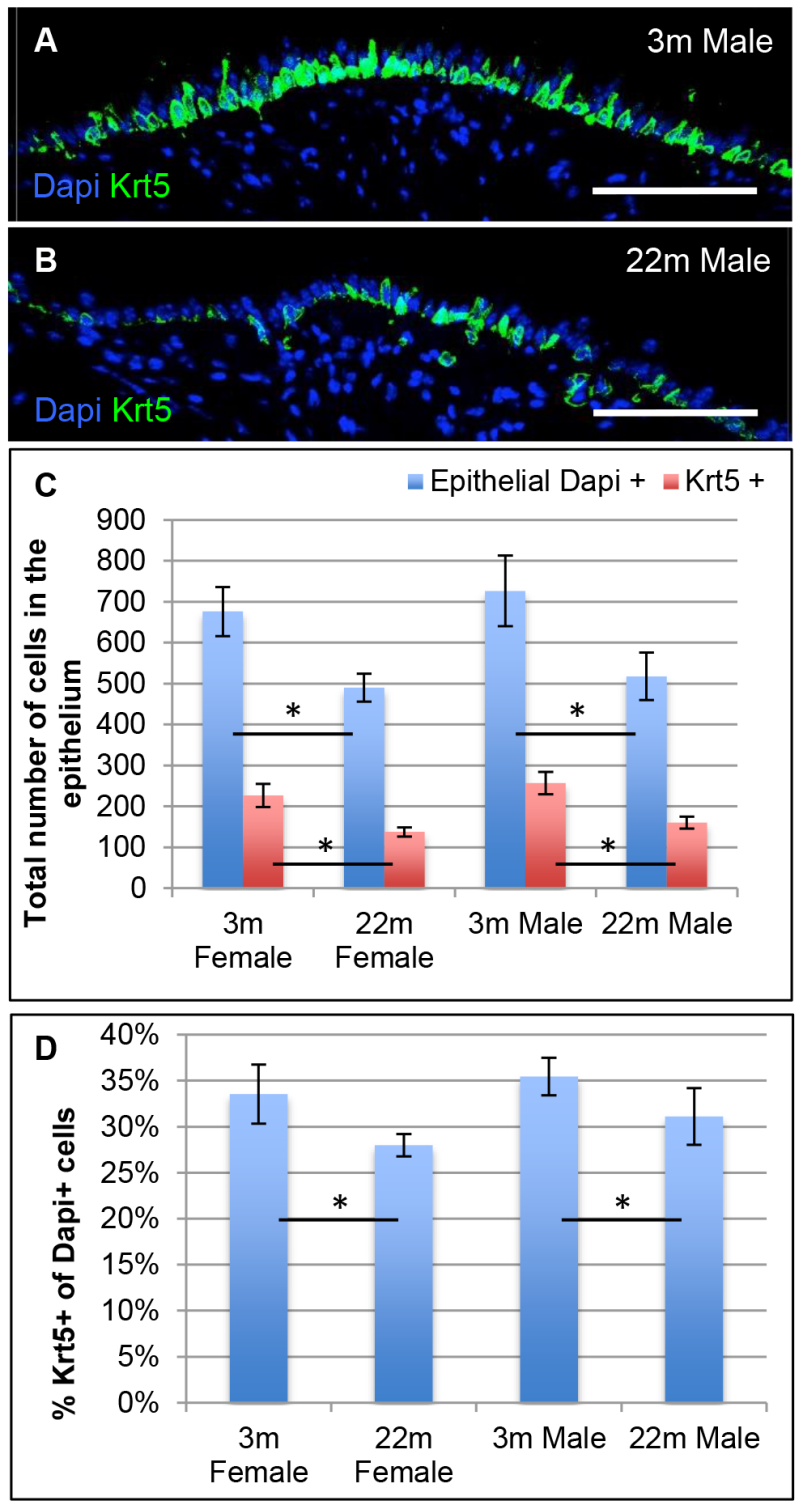
Basal cells in young and old tracheal epithelium. Tracheas from 3 young (3 month) and 3 old (22 month) male mice were fixed and midline longitudinal sections stained for nuclei (DAPI, blue) and Krt5 (green). Areas between cartilages 4 and 10 were photographed and a montage prepared. (A,B) Typical distribution of Krt5 basal cells in young versus old tracheas. (C) Total cells (blue) and total Krt5 + cells (red) present between cartilages 4 and 10. (D) Krt5+ cells as a percentage of total DAPI+ cells. The values are shown as average and SEM and p = <0.05 Scale bar: 100 um.

**Figure 4:**
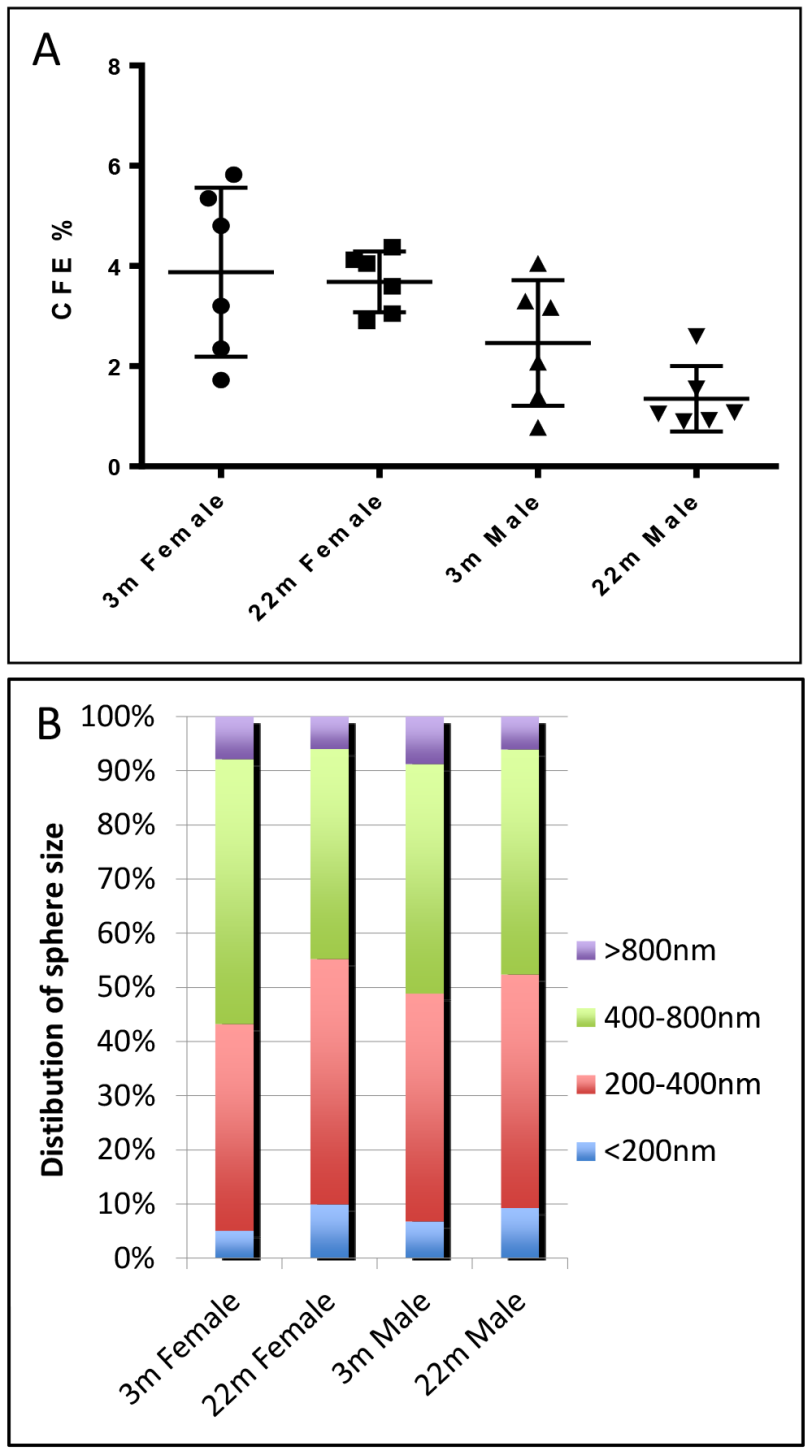
Tracheosphere assay using basal cells from young and old tracheas. NGFR+ basal cells were isolated from individual young (2 m) and old (22 m) male and female C57Bl/6 mice and seeded in quadruplicate at 1000 cells/well in Matrigel in transwell inserts. After 14 days the number of spheres were counted and the diameter measured using Fiji software. (A) Each point represents the average colony forming efficiency (CFE) of quadruplicate wells. Horizontal lines represent mean within groups, with standard deviation error bars. (B) Size distribution of spheres in all 24 wells of mice of different gender and ages.

In addition to studying basal cells in ARGLS we also analyzed the distribution of Krt5+ cells in the pseudostratified epithelium lining the trachea itself. As shown in Fig. 3A, B, there are about 25% fewer epithelial cells between cartilages 4 and 10 in older (22 month) compared with younger (3 month) mice (Fig. 3A–C). The number of Krt5+ basal cells is also reduced, as well as the proportion of the total that they represent (27 and 31% in older females and males, compared with 33 and 35% in young females and males, respectively) (Fig. 3D). Krt5+ cells as a population function as multipotent stem cells in the trachea of the adult mouse and can repair the surface epithelium after loss of luminal cells following exposure to inhaled sulfur dioxide [13], [21]. Our findings thus raise the possibility that the self-renewal and reparative ability of basal cells declines with age. We therefore tested the ability of basal cells to form clonal tracheospheres containing ciliated and secretory cells when cultured as organoids in Matrigel [13]. The reproducibility of the assay was high within the four replicates of cells isolated from the trachea of any individual mouse. However, there was considerable variability in the average colony forming efficiency (CFE) of basal cells between mice within the same age group (Fig. 4A). Given this variability we saw no significant difference in the average CFE of young and old mice of either sex, although there was a trend towards lower CFE in old male mice. There was also no difference in the distribution of sphere sizes among the experimental groups (Fig. 4B) and spheres from all groups contained both ciliated and secretory cells (data not shown).

Finally, we asked whether the tracheal epithelium of old mice was less able to undergo repair after killing luminal cells by exposure to sulfur dioxide. Failure of basal cells to repair the tracheal epithelium can lead to abnormal proliferation of the underlying stromal cells and even to tracheal stenosis [21], [22]. Although we only studied three young and three old male mice at one time after injury (7 days) and did not quantify cellular density within the surface epithelium, we saw no evidence for abnormal repair between the two groups (Fig. S1).

### Changes in gene expression profile and evidence for chronic, low-grade inflammation in aged tracheas

**Figure 5:**
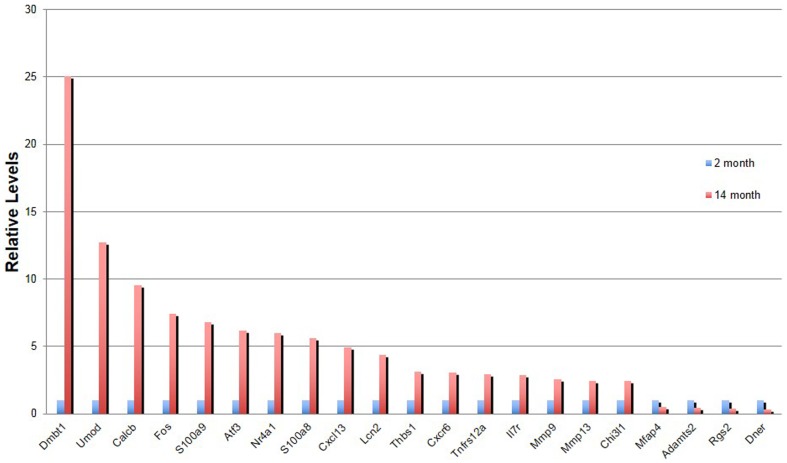
Changes in gene expression in young and old tracheas. qPCR verification of genes differentially expressed in microarray analysis. Values are average of quadruplicate samples with expression levels in 2 month old mice set to 1. Error bars represent 95% confidence interval.

Studies in several tissues and cell types (both epithelial and mesenchymal) have revealed senescence-related changes in the expression of genes encoding proteins associated with inflammation, wound healing and tissue remodeling. These changes represent either an influx of immune cells and/or a “senescence related secretory phenotype” in fibroblasts. This phenotype includes the production of growth factors, chemokines, cytokines, and interleukins that can have paracrine effects on the behavior of both immune cells and epithelial cells [23]–[25]. We therefore asked whether tracheal tissue in old mice has any senescence-related changes in gene expression and immune cell composition. We isolated total RNA from the posterior trachea and carina region of old and young female C57Bl/6 tracheas (n = 4 females at 2 and 14 months of age) and carried out Affymetrix microarray analysis. This revealed the upregulation of 87 and the downregulation of 19 genes (>2 fold, p<0.05)(Tables S1 and S2). Changes in the expression of a subset of the genes were confirmed by quantitative RT-PCR (Fig. 5). A large proportion of the differentially expressed genes (19/87), including the top ten most highly upregulated, encode immunoglobulin peptides (Table S1). Another category of differentially expressed genes encodes proteins involved in extracellular matrix composition and metabolism. This includes upregulation of transcripts for matrix metalloproteinases (MM9 and MMP13) that degrade collagen and other matrix molecules, as well as the downregulation of genes encoding collagens, elastin, fibrillin 2, Adamts2 and microfibrillar- associated protein 4 (Tables S1 and S2). Changes in collagen metabolism, including decrease in synthesis, have been reported previously in the aging rodent lung [26], [27]. One of the most highly differentially expressed non-immunoglobulin genes encodes deleted in malignant brain tumors1 protein (DMBT) (also known as hensin, gp340 and salivary agglutinin). This secreted or membrane bound protein has been implicated in innate immunity, bacterial clearance, wound healing, and response to lung infection [28]–[31].

**Figure 6:**
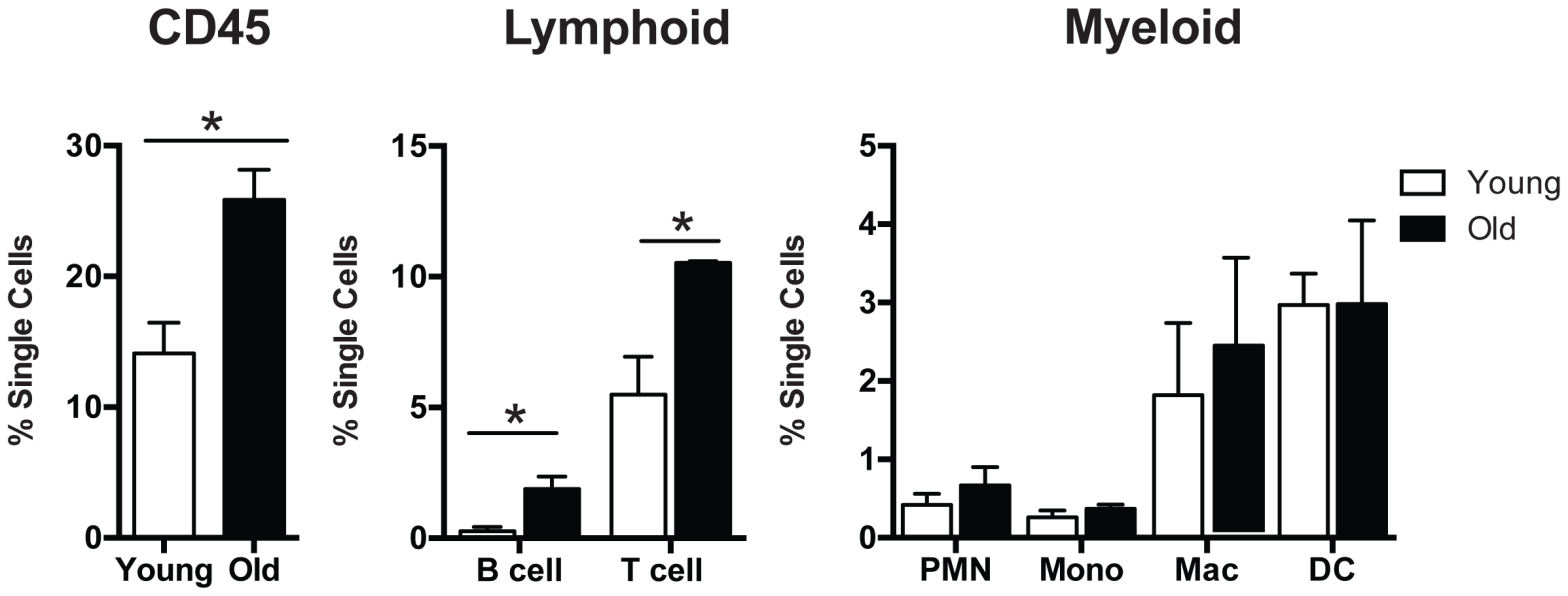
Immune cells in young and old tracheas. Tracheal cells harvested from 3 young (4 month) and three old (23 month) female C57Bl/6 mice that had only recently been imported from vendors (NIA Charles River labs in MD) were analyzed with 11 color flow cytometry. (Left panel) There was a significant increase in the percentage of CD45 cells in the aged compared with young trachea (25.87±2.30% versus 14.12±2.34%, n = 3, p<0.05). (Middle panel) There was also an increase in the percentage of cells of lymphoid lineage, such as T and B cells (p = <0.05) in old versus young tracheas. (Right panel) There was no significant change in the distribution of myeloid cell populations (polymorphonuclear leukocytes, monocytes, macrophages and dendritic cells) between the two groups.

The microarray analysis suggests that aging in the trachea is associated with an increase in local cellular immune response, including an influx of reactive B cells, possibly in response to an increase in expression of the chemokine Cxcl 13 (3.9 fold increase). In order to look for changes in immune cell composition we isolated tracheas from three young (4 month) and three older (23 month) C57Bl/6 mice, dissociated them into single cells, and analyzed the number and composition of immune cells by flow cytometry. As shown in Fig. 6 there was a significant (p<0.05) increase in the number of CD45+ cells in the tracheas of the older mice. Among the CD45+ cells there was an increase in the proportion of B and T cells but no significant change in the proportion of myeloid cells (macrophages and dendritic cells). Immunohistochemistry showed that CD45+ cells are present in the stroma underlying the tracheal epithelium and around ARGLS, especially in the intercartilage regions that are rich in blood vessels (Fig. 1H). CD45+ cells could also be seen around the small buds present at 5 months of age (Fig. 1G). However, there was no evidence for an accumulation of CD45+ cells in discrete lymphoid nodules, known in humans as bronchial associated lymphoid tissue or BALT [32], [33], in older compared with younger mice.

## Discussion

In this paper we confirm an earlier report that mice of different genetic backgrounds and bred under specific pathogen free conditions develop numerous age-related gland-like structures (ARGLS) underneath the tracheal epithelium, from around 5–7 months of age [17]. Here, we focused primarily on male and female mice of the C57Bl/6 background obtained from several commercial sources used by investigators studying aging. We saw no difference in the number and distribution of ARGLSs depending on whether the mice were examined soon after their arrival at our facility or after being maintained there for several months. Like the original authors we therefore think that the development of the cystic dysplasia is not related to acute bacterial or viral infections. However, as discussed below, low-grade tissue inflammation intrinsic to the aging process may play a role.

One of the unanswered questions about ARGLS is how they arise. One possibility was that they grow from very small or cryptic submucosal glands (SMGs) already present in the intercartilage regions. However, we show here that ARGLSs, unlike SMGs, do not have ciliated ducts and do not contain Krt5+ myoepithelial basal cells that express high levels of smooth muscle actin. Rather, both luminal and basal cells in the ARGLSs resemble those present in the tracheal epithelium, supporting the idea that the cystic structures develop de novo by budding from the surface. Furthermore, already at 5 months of age we see a few small clusters of Krt5+ cells extending from the surface epithelium into the mesenchyme in the intercartilage regions. Some of these cells express a reporter for canonical Wnt signaling, suggesting that the initial stages of ARGLS formation involve this signaling pathway. Previous studies have shown that canonical Wnt signaling is required for the initial induction and budding of SMGs [15], [16]. There is some evidence that systemic factors present in the serum of old mice increase the level of canonical Wnt signaling in muscle satellite progenitor cells [34]. If such systemic changes also affect the tracheal epithelium, additional mechanisms would have to localize the senescent signaling to the intercartilage regions since a general upregulation of the Tcf/lef-GFP reporter was not seen throughout the luminal epithelium of older animals. Further studies will be needed to explore the potential role of changes in either systemic factors, the extracellular matrix and/or the local microenvironment [35], [36] in the induction of ARGLSs from the surface epithelium.

Our microarray and immunohistochemical data provide evidence that aging results in low grade chronic inflammation in the trachea, including an increase in activated (immunoglobulin producing) B cells and T cells. Previous investigations have documented age-related changes in the accumulation of immunoglobulins and inflammatory cells in the peripheral tissues of the old lungs of DBA/2 mice but this analysis did not extend to the trachea and main stem bronchi [7]. Other studies on the aging mouse lung also did not include the trachea and extralobar airways [37]. Neither the cause nor the functional significance of the immunomodulation we have observed in the trachea of the older mouse lung is clear at this time. We failed to see premature budding or delamination of the epithelium after multiple rounds of injury and repair caused by exposure to inhaled SO_2_. We also did not see nascent ARGLS in young transgenic mice overexpressing epithelial Na+ channel beta subunit in airway epithelium, a condition associated with neutrophilic inflammation [38] or in mice repeatedly infected with Mycoplasma pneumonia [39] (see Materials and Methods for experimental details). These results suggest that ARGLS do not form in response to acute inflammation but may reflect a response of the mucociliary epithlium to chronic changes in the aging tissue microenvironment [1]. Further study will also be required to determine the cause of the reduced number and proportion of basal cells that we have documented in the tracheal epithelium of old mice. In both cased the studies are likely to be relevant to the human lung in which a pseudostratified epithelium with basal cells is present throughout most of the intralobar conducting airways.

## Materials and Methods

### Mice

Male and female C57Bl/6 mice were obtained from three sources: The Jackson Laboratory (6 months old) and Charles River Laboratories Inc. located at either Frederick, Maryland (NCI, 4 weeks to 6 months old) or Gaithersberg, Maryland (NIA, 3 month and 22 month old). Following import they were maintained under identical high-level barrier conditions in one room of the animal facility of Duke University Medical Center (DUMC). Only animals that meet specific health standards are housed in this room, in individually ventilated cage rack systems. Caging and bedding are autoclaved, food is either autoclaved or irradiated, and cages are supplied with reverse osmosis purified water by an automatic watering system. All cage changing and procedures are conducted under a HEPA filtered cage-changing station or class II BSC. *Myd88^tm1Aki^* null mice on the C57Bl/6 background were originally from The Jackson laboratory and were maintained at DUMC and kindly provided by Paul Noble. Transgenic mice expressing epithelial Na+ channel beta subunit in airway epithelium (*Tg(Scgb1a1-Scnn1b)6608Bouc* on C57Bl/6 background [38]) were kindly provided by Scott Randell, University of North Carolina Chapel Hill. Canonical Wnt signaling reporter mice (*Tg(TCF/Lef1-HIST1H2BB/EGFP)61Hadj/J*) were a kind gift from Anna-Katerina Hadjankonatis, Memorial Sloan-Kettering Cancer Center, New York [18]. Homozygous female *Ob/Ob* mice (*B6.Cg-Lep0b/J*) (20 weeks old, n = 2) were from the Jackson Laboratory.

### Injury and infection models

Three young and three old male mice were exposed once to 500 ppm SO2 for four hours and tracheal morphology examined after 24 hrs and 7 days, as previously described [40]. In another case three 8 week old C57Bl/6 male mice were given five rounds of exposure to 500 ppm SO_2_. Each exposure was for 4 hours followed by 2 weeks of repair. Infection with *Mycoplasma pneumoniae* (Mp) from the American Type Culture Collection (Cat Number 15531) was as described [39]. Male C57Bl/6 mice (8–10 weeks old n = 4) were treated intranasally on day 0 with 1×10^8^ Mp under anesthesia. Mice were treated again on days 7, 14 and 21 with 1×10^6^ Mp.

### Histology, Immunohistochemistry and Flow analysis

Tracheas were fixed with 4% paraformaldehyde in PBS and either embedded in paraffin or OCT before serial sectioning at 7 μm. Antigen retrieval was performed using 10 mM sodium citrate in a 2100 Antigen Retriever (Aptum Biologics Ltd.). Sections were subsequently stained using the following antibodies: mouse anti acetylated tubulin, 1∶1000 (Sigma); chicken anti GFP, 1∶500 (Aves Labs); rabbit anti Keratin5, 1∶500 (Convance); mouse anti Keratin14, 1∶100 (Thermo Scientific Lab Vision); rabbit anti Keratin14, 1∶500 (Covance); rabbit anti Lactotransferrin, 1∶5000 (Millipore), mouse anti alpha Smooth muscle actin, 1∶200 (Sigma), rat anti Keratin8/Troma 1c, 1∶100 (Developmental Studies Hybridoma Bank). Alexa488, Alexa555 and Alexa647 secondary antibodies were from Molecular Probes. Sections were analyzed using a Zeiss 710 inverted confocal microscope system. For quantification of Krt5+ basal cells midline sections were selected from 3 young (3 month) and 3 older (22 month) male and female mice, stained with antibodies to Krt5, Krt8, acetylated tubulin and DAPI and photographed between cartilages 4 and 10 using a Zeiss Axio Imager D2 microscope. Total Krt5+ cells were counted along both lateral surfaces and calculated as a percentage of total DAPI+ cells. ARGLS and epithelial cells below the surface epithelium were not included.

In situ hybridization was performed as described by Wansleeben et al. 2010 [41].

For analysis of immune cells tracheas were harvested, cleaned of attached connective tissue, and digested with 1.5 mg/ml Collagenase A (Roche, Indianapolis, IN), 0.4 mg/ml DNase I (Roche, Indianapolis, IN), and 2 U/ml Dispase II (Sigma-Aldrich, St. Louis, MO) in Hanks balanced salt solution (Gibco) with 3% FBS and 10 mM HEPES pH 7.2–7.5. Single cell suspensions were washed and approximately, 5×10^5^ cells per trachea used for 11 color flow cytometry. Antibodies used included the following: CD45, CD11c, and IA/IE (eBioscience, San Diego, CA), CD11b and Ly6G (BD Biosciences, San Jose, CA), and F4/80, CD64, CD24, and CD31 (Biolegend, San Diego, CA). At least one channel was utilized for detecting autofluorescence. In addition, Invitrogen Aqua Live/Dead (Grand Island, NY) was used to exclude dead cells. Data were collected with a BD LSRII flow cytometer and analyzed with Flowjo software.

### Microarray analysis

Total RNA from distal tracheas and carinas of four young (2 month) and four older (14 month) C57Bl/6 female mice was extracted using QIAshredder and RNeasy Micro Kits (QIAGEN). The quality was checked with a 2100 Bioanalyzer (Agilent Technologies). Total RNA was processed using Ambion MessageAmp Premier by the Duke Microarray Facility. Standard Affymetrix protocols and Affymetrix GeneChip Mouse Genome 430 2.0 Array chips were used to generate cel files. Data analysis was performed using Genomics Suite 6.5 (Partek) software and robust multichip analysis normalization was performed on each dataset. Two-way ANOVA and fold-change analyses were performed to select genes that were differentially expressed between 2 month and 14 month old C57Bl/6 datasets. Top differentially expressed genes were selected with a p-value cutoff of <0.05 based on ANOVA test and a fold-change cutoff of >±2.0. The NCBI GEO accession number for the microarray data is GSE55162.

### qRT-PCR analysis

Total RNA from each of the four biological replicates used for the microarray was used to synthesize cDNA using iScript cDNA Synthesis Kit (Bio-Rad). Gene expression levels were quantified by qRT-PCR on the StepOnePlus Real-Time PCR System (Applied Biosystems). Threshold cycle values (Ct) for samples were normalized to GAPDH (ΔCt), and these values across samples were compared (ΔΔCt) to quantify relative expression. Bars represent average relative expression, with expression levels in 2 month old mice set to 1. Error bars represent 95% confidence interval. Primers are listed in Table S3.

### In vitro culture of basal cells

Ngfr+ basal cells [13] were suspended in MTEC/plus medium [42], mixed 50∶50 with growth factor-reduced Matrigel (BD biosciences), and seeded at 1000 cells/well in 24 well 0.4 μm pore size transwell inserts (Falcon). Samples were prepared in quadruplicate from a single trachea of a total of 6 young (3 month) and 6 older (22 month) C57Bl/6 mice. Medium in the lower well was changed every other day. Medium was supplemented with 10 μM Rock inhibitor (Sigma, Y-27632 dihydrochloride) during first 2 days of culture. MTEC/SF [42] was used from day 7. At day 14 images were taken using AxioVert 200 M with a 1.25x objective (Carl Zeiss) that allows imaging of the entire well. The total number of spheres was counted and the diameter measured using Fiji software. Data points in Fig. 4A represent average CFE of quadruplicate wells of cultures of each individual mouse. Horizontal lines represents mean within groups with standard deviation error bars.

### Ethics statement

Experiments with mice were approved by the Duke University Institutional Animal Care and Use Committee under protocol A262-13-10.

## Supporting Information

Figure S1 **Repair of tracheal epithelium after loss of luminal cells.** Sections of tracheas of young (3 month) and old (22month) male mice 7 days after exposure to sulfur dioxide. Sections were examined by immunohistochemistry (A, B) and haematoxylin and eosin staining (A′, B′). Similar results were seen in two other mice in each group. Staining of sections 24 hrs after exposure confirmed that the extent of damage to luminal cells was comparable in young and old mice Scale bars 50 um.
(TIF)

Table S1 **Genes differentially expressed at more than 2 fold higher levels in tracheas of old (14 month) versus young (2 month) mice (p<0.05).**
(DOCX)

Table S2 **Genes differentially expressed at more than 2 fold lower levels in tracheas of old versus young mice.**
(DOCX)

Table S3 **Primers for qPCR analysis.**
(DOCX)
