# Supplementary figures and images for: Age-Related Changes in the Cellular Composition and Epithelial Organization of the Mouse Trachea
Source: PLoS One. 2014 Mar 27;9(3):e93496. doi: 10.1371/journal.pone.0093496 (PMC3968161; doi:10.1371/journal.pone.0093496)

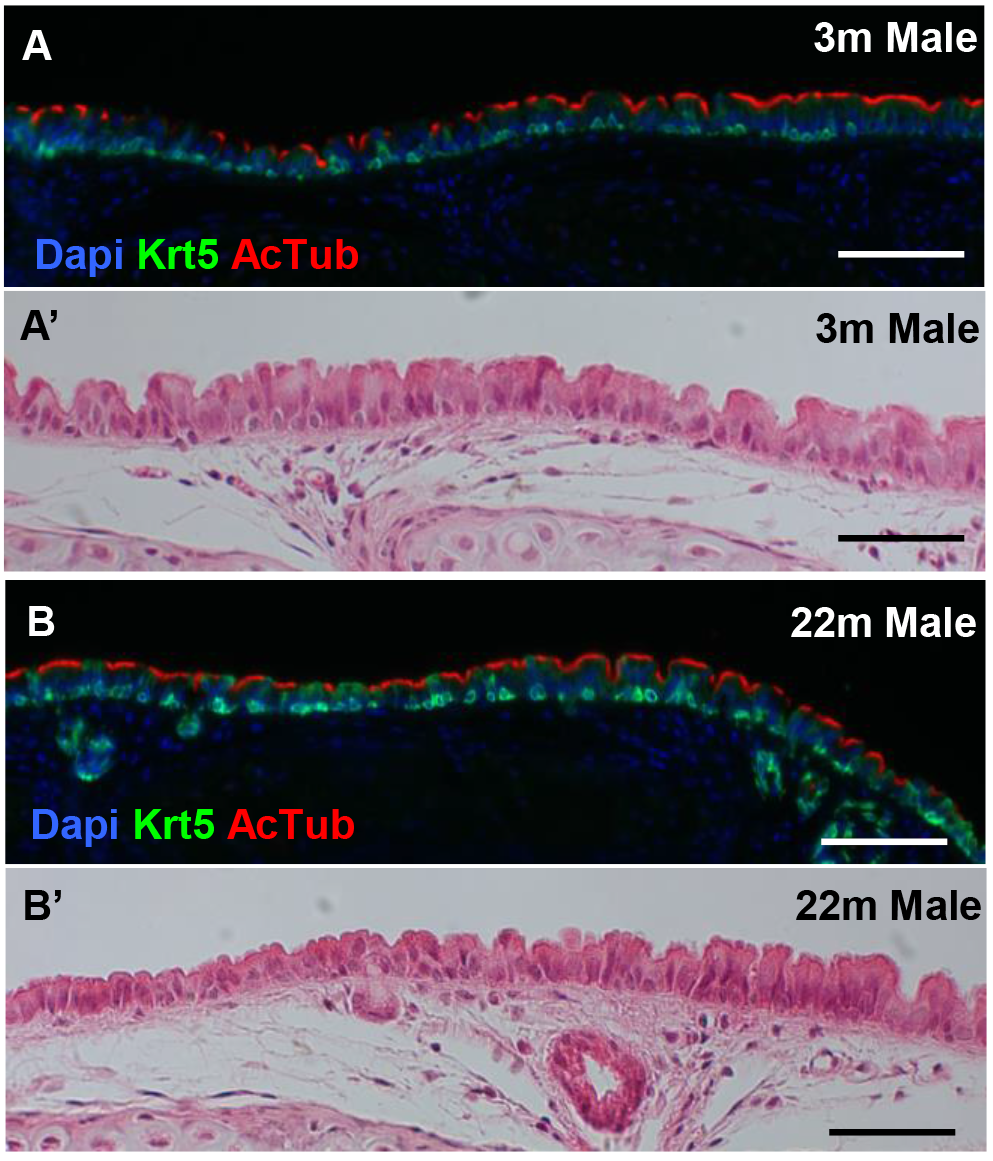

Supplement: Figure S1 — Repair of tracheal epithelium after loss of luminal cells. Sections of tracheas of young (3 month) and old (22month) male mice 7 days after exposure to sulfur dioxide. Sections were examined by immunohistochemistry (A, B) and haematoxylin and eosin staining (A′, B′). Similar results were seen in two other mice in each group. Staining of sections 24 hrs after exposure confirmed that the extent of damage to luminal cells was comparable in young and old mice Scale bars 50 um. (TIF) [file pone.0093496.s001.tif]
