# Supplementary material for: Age-Related Changes in the Cellular Composition and Epithelial Organization of the Mouse Trachea
Source: PLoS One. 2014 Mar 27;9(3):e93496. doi: 10.1371/journal.pone.0093496 (PMC3968161; doi:10.1371/journal.pone.0093496)
Supplement: Table S1 — Genes differentially expressed at more than 2 fold higher levels in tracheas of old (14 month) versus young (2 month) mice (p<0.05). (DOCX) [file pone.0093496.s002.docx]

| **Gene Symbol** | **Gene Title** | **Fold-Change** |
| --- | --- | --- |
| Gm5571 /// Igk-V1 | predicted gene 5571 /// immunoglobulin kappa chain variable 1 (V1) | 46.6964 |
| Igj | immunoglobulin joining chain | 24.7376 |
| 2010309G21Rik /// Igl-C2 | RIKEN cDNA 2010309G21 gene /// immunoglobulin lambda chain, constant region 2 | 23.4103 |
| Gm10883 /// Gm1420 /// Gm7202 /// Igk-C /// Igk-J1 /// Igk-V28 | predicted gene 10883 /// predicted gene 1420 /// predicted gene 7202 /// immunoglobulin | 21.8056 |
| 2010309G21Rik /// Igl-C2 /// Igl-C3 | RIKEN cDNA 2010309G21 gene /// immunoglobulin lambda chain, constant region 2 /// immunoglobulin | 18.3222 |
| Igh-6 | immunoglobulin heavy chain 6 (heavy chain of IgM) | 16.2412 |
| Igl-V1 | immunoglobulin lambda chain, variable 1 | 15.8685 |
| Dmbt1 | deleted in malignant brain tumors 1 | 15.1127 |
| Igh-2 /// Igh-VJ558 /// LOC544903 | immunoglobulin heavy chain 2 (serum IgA) /// immunoglobulin heavy chain (J558 family) / | 14.3794 |
| Igh-2 /// Igh-VJ558 /// LOC544903 | immunoglobulin heavy chain 2 (serum IgA) /// immunoglobulin heavy chain (J558 family) / | 14.1458 |
| Igh-2 /// Igh-VJ558 /// LOC544903 | immunoglobulin heavy chain 2 (serum IgA) /// immunoglobulin heavy chain (J558 family) / | 13.5752 |
| Igh-6 | immunoglobulin heavy chain 6 (heavy chain of IgM) | 12.9682 |
| Gm10883 /// Gm1420 /// Gm7202 /// Igk-C /// Igk-J1 /// Igk-V28 | predicted gene 10883 /// predicted gene 1420 /// predicted gene 7202 /// immunoglobulin | 11.6589 |
| Gm10883 /// Gm1420 /// Gm7202 /// Igk-C /// Igk-J1 /// Igk-V28 | predicted gene 10883 /// predicted gene 1420 /// predicted gene 7202 /// immunoglobulin | 10.5904 |
| Igh-6 | Immunoglobulin heavy chain 6 (heavy chain of IgM) | 9.89929 |
| Csprs /// Gm7592 | component of Sp100-rs /// predicted gene 7592 | 9.39506 |
| Ighg | Immunoglobulin heavy chain (gamma polypeptide) | 7.19772 |
| Gm16939 | predicted gene, 16939 | 7.12142 |
| Hspa8 /// LOC624853 | heat shock protein 8 /// hypothetical LOC624853 | 5.71114 |
| Hspa1a | heat shock protein 1A | 4.52085 |
| Igh-3 /// Ighg | immunoglobulin heavy chain 3 (serum IgG2b) /// Immunoglobulin heavy chain (gamma polype | 4.50501 |
| Hspa1b | heat shock protein 1B | 4.45272 |
| Igl-V1 | Immunoglobulin lambda chain, variable 1 | 4.38103 |
| Lcn2 | lipocalin 2 | 4.24049 |
| Nr4a1 | nuclear receptor subfamily 4, group A, member 1 | 3.96556 |
| Cxcl13 | chemokine (C-X-C motif) ligand 13 | 3.94581 |
| Umod | uromodulin | 3.92522 |
| Hspa1b | heat shock protein 1B | 3.81852 |
| S100a8 | S100 calcium binding protein A8 (calgranulin A) | 3.79733 |
| Atf3 | activating transcription factor 3 | 3.50269 |
| Cyr61 | cysteine rich protein 61 | 3.36628 |
| Csprs | component of Sp100-rs | 3.27871 |
| Pou2af1 | POU domain, class 2, associating factor 1 | 3.04771 |
| Sult1c1 | sulfotransferase family, cytosolic, 1C, member 1 | 2.99493 |
| Ccl8 | chemokine (C-C motif) ligand 8 | 2.94238 |
| Thbs1 | thrombospondin 1 | 2.9164 |
| Coch | coagulation factor C homolog (Limulus polyphemus) | 2.82749 |
| S100a9 | S100 calcium binding protein A9 (calgranulin B) | 2.70078 |
| Nts | neurotensin | 2.62973 |
| Fos | FBJ osteosarcoma oncogene | 2.61046 |
| Spib | Spi-B transcription factor (Spi-1/PU.1 related) | 2.58586 |
| Tcrg-V4 | T-cell receptor gamma, variable 4 | 2.56312 |
| LOC664787 | similar to Sp110 nuclear body protein | 2.51272 |
| Cd177 | CD177 antigen | 2.48653 |
| Krt4 | keratin 4 | 2.46837 |
| Rgs13 | regulator of G-protein signaling 13 | 2.46195 |
| Tcrg-V2 /// Tcrg-V3 | T-cell receptor gamma, variable 2 /// T-cell receptor gamma, variable 3 | 2.45426 |
| Thbs1 | thrombospondin 1 | 2.4439 |
| Abca13 | ATP-binding cassette, sub-family A (ABC1), member 13 | 2.43061 |
| Mlana | melan-A | 2.42222 |
| Cd3g | CD3 antigen, gamma polypeptide | 2.416 |
| Gbp8 | guanylate-binding protein 8 | 2.38634 |
| Umod | uromodulin | 2.37559 |
| Calcb | calcitonin-related polypeptide, beta | 2.32698 |
| Expi | extracellular proteinase inhibitor | 2.31707 |
| 1500015O10Rik | RIKEN cDNA 1500015O10 gene | 2.3167 |
| Cdhr1 | cadherin-related family member 1 | 2.29764 |
| Ugt2b34 | UDP glucuronosyltransferase 2 family, polypeptide B34 | 2.29361 |
| Nr4a3 | nuclear receptor subfamily 4, group A, member 3 | 2.26516 |
| Ccl20 | chemokine (C-C motif) ligand 20 | 2.23156 |
| Mmp13 | matrix metallopeptidase 13 | 2.22448 |
| Pmaip1 | phorbol-12-myristate-13-acetate-induced protein 1 | 2.21357 |
| Ugt2b34 | UDP glucuronosyltransferase 2 family, polypeptide B34 | 2.20947 |
| Tnfrsf12a | tumor necrosis factor receptor superfamily, member 12a | 2.17685 |
| Csn2 | casein beta | 2.16691 |
| Ly6d | lymphocyte antigen 6 complex, locus D | 2.15895 |
| Il7r | interleukin 7 receptor | 2.15025 |
| Gm7202 /// Igk-C /// Igk-V19-14 /// Igk-V28 /// Igkv6-25 | predicted gene 7202 /// immunoglobulin kappa chain, constant region /// immunoglobulin | 2.14746 |
| Hbb-b1 /// Hbb-b2 /// LOC100503605 | hemoglobin, beta adult major chain /// hemoglobin, beta adult minor chain /// hemoglobi | 2.14152 |
| Cxcr6 | chemokine (C-X-C motif) receptor 6 | 2.13432 |
| Bcl2a1a /// Bcl2a1b /// Bcl2a1d | B-cell leukemia/lymphoma 2 related protein A1a /// B-cell leukemia/lymphoma 2 related p | 2.12866 |
| C2 /// Cfb | complement component 2 (within H-2S) /// complement factor B | 2.12845 |
| Egr3 | early growth response 3 | 2.11418 |
| Gm6273 /// LOC381765 /// LOC665506 /// Tcrb-J | predicted gene 6273 /// similar to T cell antigen receptor /// similar to T-cell recept | 2.10192 |
| Gm5571 /// Igk-V1 | predicted gene 5571 /// immunoglobulin kappa chain variable 1 (V1) | 2.09074 |
| Chi3l1 | chitinase 3-like 1 | 2.08033 |
| Abca13 | ATP-binding cassette, sub-family A (ABC1), member 13 | 2.08016 |
| Tcrg-V4 | T-cell receptor gamma, variable 4 | 2.07872 |
| Serpinb1a | serine (or cysteine) peptidase inhibitor, clade B, member 1a | 2.07737 |
| Ces1f | carboxylesterase 1F | 2.07416 |
| Glrb | glycine receptor, beta subunit | 2.06316 |
| Mmp9 | matrix metallopeptidase 9 | 2.05947 |
| Krt4 | keratin 4 | 2.05859 |
| Tnfrsf12a | tumor necrosis factor receptor superfamily, member 12a | 2.05706 |
| Cxcl13 | chemokine (C-X-C motif) ligand 13 | 2.04341 |
| Hsph1 | heat shock 105kDa/110kDa protein 1 | 2.04043 |
| Igh-6 | Immunoglobulin heavy chain 6 (heavy chain of IgM) | 2.0303 |
