# Supplementary material for: Age-Related Changes in the Cellular Composition and Epithelial Organization of the Mouse Trachea
Source: PLoS One. 2014 Mar 27;9(3):e93496. doi: 10.1371/journal.pone.0093496 (PMC3968161; doi:10.1371/journal.pone.0093496)
Supplement: Table S2 — Genes differentially expressed at more than 2 fold lower levels in tracheas of old versus young mice. (DOCX) [file pone.0093496.s003.docx]

| **Gene Symbol** | **Gene Title** | **Fold-Change** |
| --- | --- | --- |
| Adamts2 | a disintegrin-like and metallopeptidase (reprolysin type) with thrombospondin type 1 motif | -3.00324 |
| Eln | elastin | -2.86545 |
| Col3a1 | collagen, type III, alpha 1 | -2.61657 |
| Col1a1 | collagen, type I, alpha 1 | -2.47006 |
| Bex1 | brain expressed gene 1 | -2.41309 |
| Col10a1 | collagen, type X, alpha 1 | -2.37229 |
| Col1a2 | collagen, type I, alpha 2 | -2.33816 |
| Rgs2 | regulator of G-protein signaling 2 | -2.27256 |
| Dbp | D site albumin promoter binding protein | -2.21659 |
| Cilp | cartilage intermediate layer protein, nucleotide pyrophosphohydrolase | -2.1899 |
| Fbn2 | fibrillin 2 | -2.17992 |
| Apobec2 | apolipoprotein B mRNA editing enzyme, catalytic polypeptide 2 | -2.17907 |
| Otor | otoraplin | -2.16173 |
| Dner | delta/notch-like EGF-related receptor | -2.16035 |
| Peg3 | paternally expressed 3 | -2.13206 |
| Cckar | cholecystokinin A receptor | -2.10543 |
| A2m | alpha-2-macroglobulin | -2.09428 |
| Synpo2l | synaptopodin 2-like | -2.04692 |
| Mfap4 | microfibrillar-associated protein 4 | -2.00261 |
