# Supplementary material for: Age-Related Changes in the Cellular Composition and Epithelial Organization of the Mouse Trachea
Source: PLoS One. 2014 Mar 27;9(3):e93496. doi: 10.1371/journal.pone.0093496 (PMC3968161; doi:10.1371/journal.pone.0093496)
Supplement: Table S3 — Primers for qPCR analysis. (DOCX) [file pone.0093496.s004.docx]

| **Primer** | **Sequence** |
| --- | --- |
| Dmbt1_Fw | TGGAGGCTATGAGGACTATCTG |
| Dmbt1_rev | TGGTTTGGTCAGTTGGGTAG |
| Lcn2_Fw | CTACAATGTCACCTCCATCCTG |
| Lcn2_rev | ACCTGTGCATATTTCCCAGAG |
| Nr4a1_Fw | GCCTAGCACTGCCAAATTG |
| Nr4a1_Rev | TCTGCCCACTTTCGGATAAC |
| Cxcl13_Fw | AGATCGGATTCAAGTTACGCC |
| Cxcl13_rev | ACAGACTTTTGCTTTGGACATG |
| Umod_Fw | CTTACTGCACCGATCCTAGTTC |
| Umod_rev | CTCCAGCCTGTACTCCAATTG |
| S100a8_Fw | AGTGTCCTCAGTTTGTGCAG |
| S100a8_rev | ACTCCTTGTGGCTGTCTTTG |
| Atf3_Fw | ATAAACACCTCTGCCATCGG |
| Atf3_rev | GCCTCCTTTTCCTCTCATCTTC |
| Pou2af1_Fw | CACGCCCAGTCACATTAAAG |
| Pou2af1_rev | GACTCGAACACCCTGGTATG |
| Ccl8_Fw | AGGGATTGAGAGGACGCTAG |
| Ccl8_rev | GGTGACTGGAGCCTTATCTG |
| Thbs1_Fw | TCCCCTCTGCTTTCACAATG |
| Thbs1_rev | TCAGGAACTGTGGCGTTG |
| S100a9_Fw | GCACAGTTGGCAACCTTTATG |
| S100a9_rev | CTCATGACAGGCAAAGATCAAC |
| Fos_Fw | CTGAAGAGGAAGAGAAACGGAG |
| Fos_rev | CAATCTCAGTCTGCAACGC |
| Calcb_Fw | GCACGATATGGGTCCTGTG |
| Calcb_rev | CCTTCATCTGCTCATAGTCCTG |
| Cdhr1_Fw | GGACAGAGAAAGGGAAGATGAG |
| Cdhr1_rev | ATGTAAGGCTCCTGGATGAAC |
| Ccl20_Fw | AAGACAGATGGCCGATGAAG |
| Ccl20_rev | TCTTGACTCTTAGGCTGAGGA |
| Mmp13_Fw | GATTATCCCCGCCTCATAGAAG |
| Mmp13_rev | TCTCACAATGCGATTACTCCAG |
| Tnfrsf12a_Fw | GATTCGGCTTGGTGTTGATG |
| Tnfrsf12a_rev | CAGGCAGAAGTCGCTGTG |
| Csn2_Fw | CAGAAGGTGAATCTCATGGGAC |
| Csn2_rev | AGGCTGGATGTTTTGTGGG |
| Il7r_Fw | TCTGGAGAAAGTGGAAATGCC |
| Il7r_rev | AGCTGTGTTGATGTCTGAGTC |
| Cxcr6_Fw | AGCACACTTCACTCTGGAAC |
| Cxcr6_rev | TTGAAGAGCCAGAAATCTCCC |
| Chi3l1_Fw | AGAAACACCAACCTGAAGACC |
| Chi3l1_rev | CCCATCAAAGCCATAAGAACG |
| Mmp9_Fw | GATCCCCAGAGCGTCATTC |
| Mmp9_rev | CCACCTTGTTCACCTCATTTTG |
| Cxcl13_Fw | AGATCGGATTCAAGTTACGCC |
| Cxxl13_rev | ACAGACTTTTGCTTTGGACATG |
| Mfap4_Fw | TCAACGGCTCAGTGAGTTTC |
| Mfap4_rev | AAGTCTTCCAAGTCCACGC |
| Cckar_Fw | GACTCCGTACCCCATTTACAG |
| Cckar-rev | GAAGAGGATGAGTAGCAGGAATG |
| Dner_Fw | CGGGAAAAGGAAAATGTGCC |
| Dner_rev | ATTTGCATCAATACAGCTCGC |
| Rgs2_Fw | CTGAGAATGCAAAGTGCCATG |
| Rgs2_rev | AAGTAGCTCAAACGGGTCTTC |
| Adamts2_Fw | CAGTGGGACCTGTACTTTGAG |
| Adamts2_rev | ACCATGCGCTTCATAGACAC |
